# Sex and age-dependent characterization of the circadian clock as a potential biomarker for physical performance: A prospective study protocol

**DOI:** 10.1371/journal.pone.0293226

**Published:** 2023-10-24

**Authors:** Müge Yalçin, Angela Relógio

**Affiliations:** 1 Institute for Theoretical Biology (ITB), Charité—Universitätsmedizin Berlin, corporate member of Freie Universität Berlin, Humboldt-Universität zu Berlin and Berlin Institute of Health, Berlin, Germany; 2 Institute for Systems Medicine and Faculty of Human Medicine, MSH Medical School Hamburg, Hamburg, Germany; Federal University of Rio Grande do Sul: Universidade Federal do Rio Grande do Sul, BRAZIL

## Abstract

**Introduction:**

Circadian rhythms (CR) regulate daily cycles in behavior, physiology and molecular processes. CRs are endogenous and vary across individuals. Seasonal changes can influence CR. Accordingly, rhythms with different characteristics (amplitude, phase) are depicted during the summer months, as compared to winter. Increasing evidence points to an influence of circadian regulation on physical performance. Here, we aim to obtain a comprehensive circadian gene expression profile for physically active individuals, which can potentially be used for the identification of optimal time intervals for physical exercise.

**Methods and analysis:**

To explore these different aspects, we propose a study where we will carry out a molecular analysis of CR by measuring the expression of specific clock and clock-controlled genes, based on a non-invasive approach using RNA extracted from saliva in physically active, healthy participants. We will collect data across two seasons and use computational algorithms to integrate the molecular data with hormonal data (cortisol and melatonin), and generate a profile of CR in healthy individuals of different sex and age groups. Finally, we will use computational tools to predict optimal time intervals for physical performance based on the above-described data, thereby retrieving valuable data on the circadian clock as a key factor for health maintenance and optimization.

## Introduction

In mammals, the time of physiological and cellular processes is regulated by an endogenous time-generating mechanism—the circadian clock, which enables optimal adaptation to external cues such as the day/night cycles [1]. The circadian system consists of a central pacemaker, located in the suprachiasmatic nucleus that synchronizes peripheral oscillators in each cell of the body. Circadian rhythms (CR) are 24-hour oscillations found in more than 40% of all protein coding genes across mammalian tissues [2]. CR are generated by a network of regulatory elements, the core-clock genes, including *BMAL1* (Basic Helix-Loop-Helix ARNT Like 1, also known as *ARNTL)*, *NR1D1*, *2* (Nuclear Receptor Subfamily 1 Group D Member 1, 2), *ROR A*, *B*, *C* (RAR-Related Orphan Nuclear Receptor A, B, C), *PER1*, *2*, *3* (Period 1, 2, 3) and *CRY1*, *2* (Cryptochrome 1, 2), interconnected via transcriptional/ translational feedback loops [3]. Both the core-clock genes, as well as its targets, the so-called clock-controlled genes (CCGs), are involved in crucial processes in the organism, including sleep/wake cycles, mood, memory formation, metabolism, and immunity [4–6].

Circadian dysregulation may occur as a result of life style factors, such as constant shift work [7, 8], or as a result of alterations in core-clock genes (due to mutations or aberrant gene expression activity) [9]. Such dysregulations are associated with several pathologies, ranging from circadian rhythm sleep disorders, under G47.20 code by International Classification of Diseases 10th Revision (ICD-10) [10], Alzheimer’s Disease and other dementias [11], Parkinson’s Disease [12], and cancer [13–15]. Hence, the study of the circadian clock in health and disease prevention is timely, and has enormous medical relevance.

The assessment of a dysregulated clock and its implication in disease requires the characterization of a healthy clock. The CR varies among individuals [16] and additional features such as sex, age or environmental influences (e.g., seasons) need to be considered. Females tend to exhibit an earlier circadian peak phase as compared to males, and previous reports showed a different therapeutic impact among females and males in colorectal cancer patients treated under chrono-modulated therapy regimes [17, 18]. Aging may also influence the CR resulting in dampening of the circadian amplitude, an advance of circadian phase, or result in gain or loss of rhythmic circadian expression [19–24]. Recent transcriptomics studies further revealed differential regulation of circadian gene expression between middle/older aged individuals, and younger adults [23, 25]. CR also fluctuate as a result of seasonal changes in light exposure as measured in sleep onset/offset times and duration [26, 27].

Monitoring the CR is possible via different methods, depending on the particular aim of the study, ranging from questionnaires to hormonal assessment via cortisol or dim-light melatonin onset [28–30]. These methods however, do not assess the molecular clock, or are time consuming and require medical assistance. Thus, a user-friendly and non-invasive method to assess the CR is advantageous. In this regard, the use of saliva-based methods, like TimeTeller^®^ [31] used in this study allows to assess gene expression profiles [31–33], and can be used to monitor the CR in humans [31–34]. The system-level regulation of the clock imposes optimal time frames for daily activities. Published data indicate an influence of circadian regulation on the physical performance for example, for professional athletes [35–37].

In this study, we aim to characterize and monitor the circadian clock with respect to sex in healthy individuals for different age groups, including teenagers (≤18 years old); younger adults (18–40 years old) and older (>40–60 years old individuals across the year via measurement of core-clock and CCGs in saliva samples. We will analyze participants with respect to additional factors based on characteristics like the chronotype, mid-sleep time, and activity scores, or frequency of sick-related absence at work, to explore a potential correlation among these different factors and gene expression changes of circadian genes. In addition, to characterize a healthy CR, we aim to combine such data to obtain a comprehensive profile of optimal performance for participants, which can potentially be generalized to other individuals with similar CR profiles. We will complement our analysis with other physiological parameters including hormonal data from melatonin, which has been reported to depict high values during the night thereby promoting sleep, and cortisol with high levels in the morning, promoting alertness. Given the increase usage of wearables and its potential for assessment of daily oscillations [38], we will also collect smart tracker data and monitor parameters such as activity, for each participant and search for significant correlations with the gene expression data.

## Materials and methods

### Aim of the study

The planned study aims at the measurement of circadian gene expression (by quantification of RNA) from saliva to characterize CR among different individuals, and compare the difference in CR in subgroups with respect to sex, age and across seasons.

### Study design

**Fig 1:**
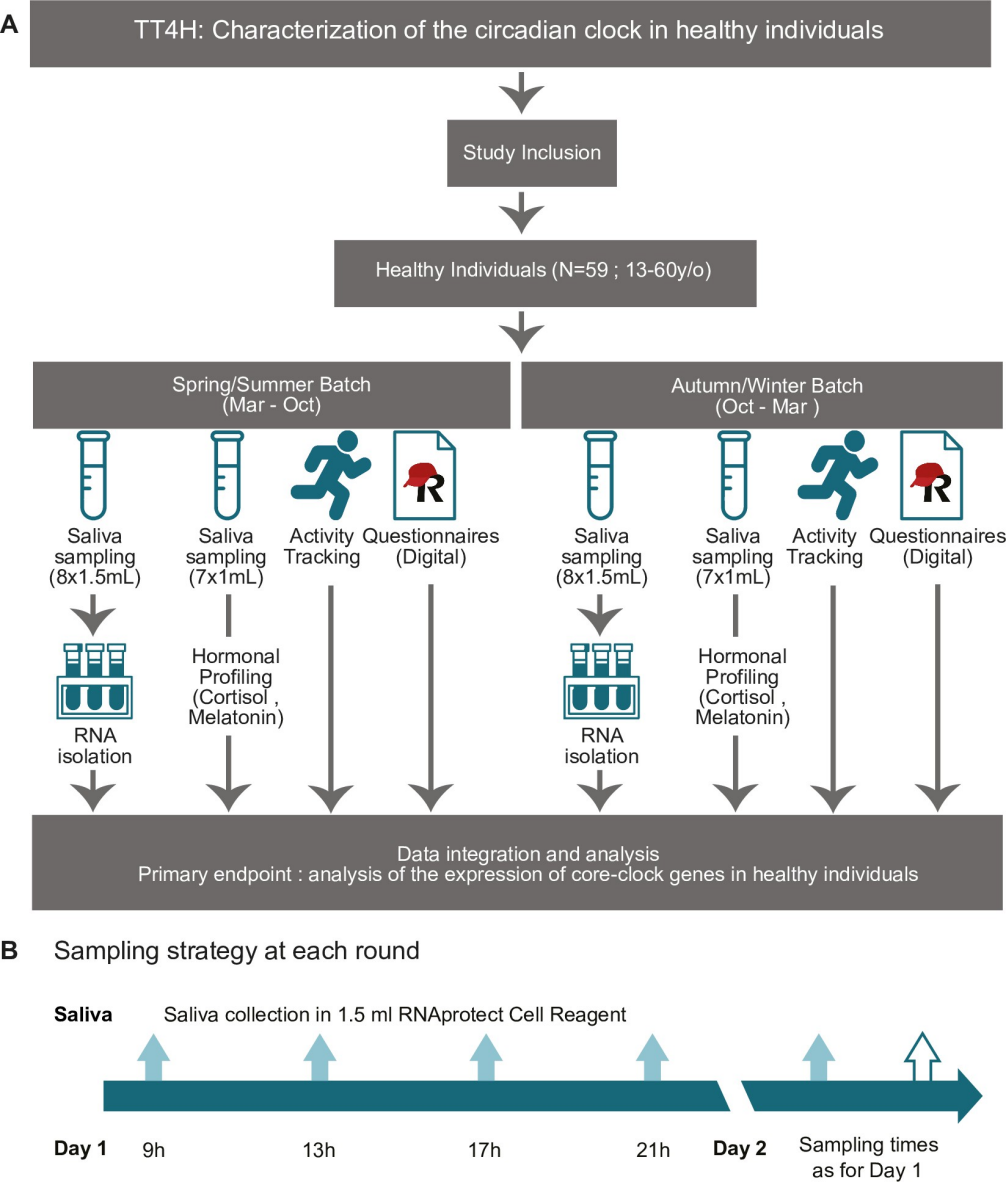
Study design flow chart. **A.** Participants meeting the study inclusion criteria will be enrolled in the study. Participants are requested to collect saliva in total for four rounds at different times of the year. Enrolled participants will fill digital forms and carry out hormonal assessment and activity tracking as indicated. **B.** Saliva sampling is carried out according to the depicted scheme. Per round of saliva sample collection, participants collect saliva four times per day, during two consecutive days (in total four rounds of saliva-sampling for CR expression will be collected per participant).

This is a prospective, monocentric, non-interventional observational study, which uses data collected from healthy and physically active participants in Germany **(Fig 1)**. Saliva samples will be collected by the participants themselves at home. For the analysis of potential seasonal variations, samples are grouped into a spring/summer batch and autumn/winter batch. Two rounds of saliva collection will take place between April-September 2022/2023 as spring/summer batch; and two rounds in October-March 2022/2023 (based on DST/standard time change date), as autumn/winter batch. In total four sampling rounds per person, till the end of 2023, are aimed to be collected. For each sampling round, 8 saliva samples per sampling kit, at four time points per day, should be collected during two consecutive days **(Fig 1)**.

### Objectives

#### Primary objective

The primary objective of the study is the molecular characterization of the individual CR in physically active, healthy volunteers (see ‘*Participant Recruitment’* section for further details) through the use of RNA extracted from biological samples (saliva), and the development of a characteristic CR profile in specific subgroups defined by biological sex, age and seasons. Although the CR varies between individuals, according to our sample size estimation, the population that is physically active and without any chronic disease at the time of sampling may therefore represent a healthy control group of participants. Comparison of variation coefficient between CR of females vs males, detection of the seasonal influence and impact of age on the CR will be evaluated.

#### Secondary objectives

Using the collected activity tracker data in parallel to the saliva sampling, a correlation analysis will be carried out between CR and the activity data. An additional subgroup analysis based on the questionnaire and medical history results and gene expression changes will be carried out. For the analysis, we plan to also include data from our previous study (Basti *et al*. 2021 [34]) in which participants carried out physical activity tests at different times of the day. This data set will be used to support the development of predictors for establishment of the optimal time intervals for physical exercise.

#### Explorative objectives

Investigation of the possible association between CR and the expression of up to 800 target genes using a multiplexing system (using a nanostring nCounter SPRINT Profiler) will be performed on a subset of individuals to allow a comparison between different age groups within the cohort. The detection of the possible association between melatonin/cortisol level and the CR is an additional explorative objective.

#### Sample size estimation

Sample size calculation was performed based on *BMAL1* gene expression data from our previous work [34] assuming that all measurements follow a sinusoidal wave function $y_{i}=A\;sin\;(\omega (t_{i}+\phi ))+M+\varepsilon_{i}$, 1 ≤ i ≤ n whereby n is the total number of samples, $\omega =\frac{2\pi }{Period}$ the frequency of the sinusoidal wave, φ the phase shift, M the rhythm-adjusted mean (MESOR), and t_i_ the observed Zeitgeber time. ε_i_ is the error term for sample i. We assume ε_i_ are identically and independently distributed from $\varepsilon_{i}∼N(0,\sigma^{2})$, where σ is the noise level. The null hypothesis that there is no circadian rhythmicity (H_0_ = 0) is tested against the alternative hypothesis that a circadian rhythmicity pattern exists (H_A_≠0). Rhythmicity will be tested using the F-test which compares the fitted model against a restricted model. For details see please Cornelissen [39]. The study is considered successful if rhythmicity is detected in the measured core-clock and clock-controlled genes obtained from saliva samples. To account for multiple comparisons (comparisons between gender and age subgroups and various seasons, in total up to 10 comparisons) the type I error is set to 0.005 according to the Bonferroni correction to hold the global significance level of α = 0.05. Underlying the data of our previous study [34], an amplitude (A) of 1.11 is estimated for *BMAL1* gene expression and the standard deviation of residuals (s) is 1.36, which gives an estimate of the intrinsic effect height of r = A/s = 0.82.

**Fig 2:**
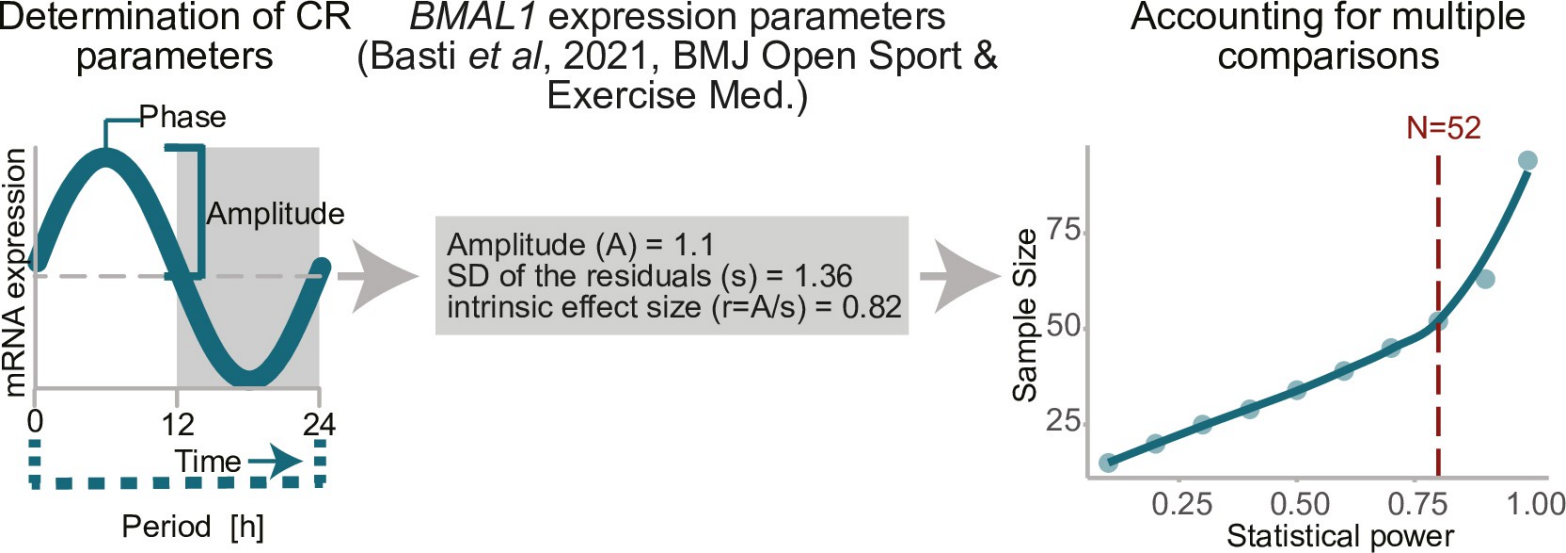
Graphical illustration of sample size estimation for the study protocol. The plot illustrates the identification of CR parameters based on *BMAL1* gene expression, which is then used to determine of sample size values and corresponding test power. Dashed red line indicates the optimal sample size (N) that needs to be collected for the detection of significant results according to pre-determined thresholds (α = 0.005; power = 0.8; r = 0.82). Accordingly, by considering a dropout rate of 12% a sample size of 59 participants was determined as needed to successfully assess the null hypothesis.

In order to detect an intrinsic effect of r = 0.82 as significant at α = 0.005 and achieve a power of 0.8, a sample size of 52 is necessary (**Fig 2**). The sample size was estimated by R package CircaPower [40]. A dropout rate of 10–15% [41–44] is generally considered for calculating the sample size in clinical studies although there is no universal filter. As a compromise for potential higher dropout due to the longitudinal design of our study, we considered a dropout of 12% in our sample size estimation, and calculated a population of 59 participants to be sufficient to allow for the planned comparisons in the subset of a) females vs males or b) age groups or c) seasons. Participants will be distributed evenly for comparisons (e.g., for sample size) or appropriate statistical measures will be taken, by using statistical tests considering unequal sample size. Participants for whom there are no saliva samples collected are considered as dropout.

### Participant recruitment

Participants will be recruited among the physically active, healthy individuals who volunteer to participate in the study and meet the inclusion criteria. Participants will be asked for their health status at the time of recruitment, which is the state of physical, mental and social well-being and not merely the absence of a chronic disease according to the definition by WHO (World Health Organization) [45]. In this study heart or respiratory diseases such as hypertension or sinusitis were not used as an exclusion criterion if participants went through treatment and were able to maintain a physically active life. For recruitment, announcements with approved flyers were distributed via the BTSC (Berlin Turn and Sports Club). Participants that are part of BTSC are either amateur or professional athletes. Participants who are not members of the sports club, will be asked whether they participate in physical activities according to the WHO definition, which includes activities such as walking, cycling, sports or active recreation and play [46].

#### Inclusion criteria

1) The consent form must be signed before study-specific tests or procedures are performed and documented. 2) Male or female participants between the ages of 13 and 60 years (inclusive) at enrollment. 3) Ability to understand and follow study-related instructions. 4) Willingness to provide saliva samples for molecular analysis.

#### Exclusion criteria

1) Participants between 13 and 18 years old without the additional consent of a legal guardian. 2) Presence of pregnancy or during breastfeeding. 3) Acute infection including oral infections.

The recruitment of participants started in May 2022 and will continue until the end of 2023.

### Data collection

#### Circadian rhythm monitoring from saliva samples

Saliva samples will be collected by the participants at home using home-kits. As a good practice for structuring circadian studies several parameters will be considered, such as sampling frequency, number of repetitive cycles or sources of biological specimen [47, 48]. For saliva collection a home kit from TimeTeller^®^ (TimeTeller GmbH, Hamburg, Germany) will be used, and sample collection carried out following the instructions of the provider, according to the previously established sampling protocol and RNA extraction and quantification [34]. During sampling, participants are advised to carry out their normal daily routine and collect a total of 8 saliva samples (with 4-hours sampling interval e.g., Day1: 9 a.m.; 1 p.m.; 5 p.m.; 9 p.m.; Day 2: 9 am; 1 p.m.; 5 pm; 9 pm) during each sampling round (**Fig 1B**). Participants are advised not to drink or eat 1 hour prior to their sampling time. The samples should be directly shipped to the lab for analysis using an enclosed package. Should this not be possible, the samples can be stored at room temperature, for a few hours or at 4°C for short-term (up to a week) or at -20°C (refrigerator) for long term storage, till shipment is possible. Further analysis including RNA quantification will be carried out by TimeTeller^®^, and the results provided in the form of individual circadian profiles.

#### Hormonal assessment

Hormonal assessment includes quantification of melatonin, a hormone that peaks at night and promotes sleep, and cortisol, which depicts higher values in the morning contributing to increased alertness. For these measurements additional saliva samples will be collected, using home-kits from cerascreen^®^ (cerascreen GmbH, Schwerin, Germany). As indicated by the provider, for melatonin 1 ml saliva will be collected in the evening before the participant goes to bed. For cortisol analysis 7 samples with 1 ml of saliva each, distributed over the course of the day, from waking up till going to bed, will be collected. The results for hormone quantification are obtained from cerascreen^®^ in the form of a report for each participant. This information can then be used to draw a correlation between collected hormonal data and gene expression obtained from saliva samples. For each participant, sampling will be repeated at least once in Spring/Summer and once in Autumn/Winter, to determine the influence of seasonal effects. To avoid possible interference with sample collection, participants are asked not to eat or drink for 30 minutes before saliva collection. In addition, it is not recommended to brush teeth or use mouthwash before sampling to avoid these interfering with the sampling.

#### Remote participant monitoring (wearable devices)

In the beginning of the study, participants will be provided a Xiaomi Smart Band 6 (MiBand 6, Xiaomi, Inc). This wrist-worn device monitors parameters such as heart rate, sleep and activity. Data from MiBand devices will be collected in real-time and synchronised with the application installed on the participant’s smartphones via Bluetooth. For this study, a cost-efficient and user-friendly smart wearable alternative was chosen, which has been previously reported to yield reasonable accuracy and precision of the outputted parameters and therefore appropriate for usage in the context of scientific research [49–51]. The participants will be advised to wear the watch at least the day before starting the saliva sampling; heart rate and sleep-wake rhythm will be recorded throughout the day. ZeppLife app will be used to export data from trackers by the participants, which will be provided to the study team using a secured folder stored at the University servers.

#### Digital questionnaires

Questionnaires and medical history will be collected by the participants in eCRF (electronic case report form) format distributed via Research Electronic Data Capture (REDCap) to preserve protected flow of information. This data will be collected at least twice within 4 total repetitive saliva sampling rounds (one in Spring/Summer and one Autumn/Winter batch maximum per participant). The general medical history is asked regarding the following: presence of known chronic or acute diseases; medications; smoking habits; presence of shift work in the past 3 months, and several questionnaires which include the μMCTQ (An Ultra-Short Version of the Munich ChronoType Questionnaire) [52] to assess the chronotype based on the core module questions of the original Munich ChronoType Questionnaire; the PSQI (The Pittsburgh Sleep Quality Index) a questionnaire to assess sleep quality [53] and the International Physical Activity Questionnaire to evaluate the physical activity patterns of participants [54], which will be used to categorize participants according to the collected questionnaire results, and may subsequently be correlated to gene expression changes. Participants will access the survey using the link provided to them, and generated using the tool REDCap, as described above, from any internet connected device (mobile or computer). Each participant can only fill their own data, during their sampling days, in pseudonymized form (using the participant codes provided by the study team), and cannot access data from another participant.

### Outcome measures

#### Primary outcome measure

Circadian profiles of gene expression from saliva samples of healthy, physically active individuals to characterize the CR. The participants will be provided four saliva sampling home kits with which they can collect their data during the four different rounds (each round of sampling collection carried out over two consecutive days) within the suggested season (spring/summer or autumn/winter) batch, see section ‘*Study Design’* for further details. Time series analysis of 8 core-clock and clock-controlled genes, *BMAL1*, *PER2*, *CRY2*, *NR1D2*, *AKT1*, *SIRT1*, *IL6*, *PDHB*, *GAPDH* (Glyceraldehyde-3-Phosphate Dehydrogenase housekeeping gene/internal reference gene), will be measured to quantify CR characteristics with respect to phase, amplitude and mesor.

#### Secondary outcome measures

Analysis of the influence of sex on the CR by comparison of CR profiles of males and females. Investigation of the seasonality of CR by collection of saliva samples during two seasons (spring/summer and fall/winter). Analysis of the influence of age on the CR by comparison of CR profiles of younger probands (age between 13 and 18 years old) and profiles of older and younger adults, considering participants between 18–39 and 40–60 years old in separate groups.

#### Explorative outcome measures

Additionally, clock and clock-related genes will be measured using a nanostring nCounter SPRINT Profiler using gene expression panels (Homo Sapiens Metabolic Pathways and a custom designed panel based on Extended Core Clock Network [55]) in a subset of participants to allow a better comparison of aging associated alterations of CR expression. By hormonal measurement at two different times of the year, the correlation between core-clock gene expression, cortisol and melatonin measurements will be investigated. The correlation between circadian gene expression and physiological parameters (daily activity measurements, heart rate, sleep) obtained by fitness trackers provided to the participants, will be explored.

### Data management

The study team will archive the source documents for each participant, which consists of the informed consent forms, saliva sample collection form, electronic participant surveys, reports for melatonin concentrations and cortisol profile, exported activity data from fitness tracker and gene expression results. The questionnaire and medical history data are collected by the participants themselves using the REDCap application [56]. In addition, the participants are asked to collect information on the exact times of the saliva sampling and time of daily habits, such as wake/sleep, and meal times during the sample collection days. Each participant is provided a pseudonymized self-reporting sheet for this purpose.

The study will be carried out in accordance with the applicable data protection regulations. All personal data collected during the study is pseudonymized before it is further conveyed, i.e., the recipient cannot establish a connection between the data and the participant. For this purpose, each participant is assigned a 12-digit randomized number as participant ID, generated using the R sample() function. The identifying "pseudonym keys" are stored in a re-identification list and only accessible to the study management. All data will only be communicated and published in anonymized way so that it will not be possible to draw any conclusions regarding the identity of the subject. The pseudonymously personal reference to the clinical data will be deleted after 10 years of archive period.

### Safety considerations

This study is a non-interventional, observational study therefore a safety consideration plan is not applicable. All procedures involving human subjects were approved by Charité -Universitätsmedizin Berlin Ethics Committee (EA2/242/20).

### Statistical analysis plan

#### Descriptive analyses

All continuous variables will be summarized using the following descriptive statistics: n (non-missing sample size), mean, standard deviation, median, maximum and minimum. The number and percentages (based on the non-missing sample size) of observed levels will be reported for all categorical measures. All summary tables will be structured with a row for each point in time and subgroup (if relevant) and will be annotated with the total population size relevant to that table/subgroup, including any missing observations.

#### Analysis for primary endpoint

Amplitude, phase and mesor of the circadian data for the subgroups according to sex, age and season will be estimated per core clock gene using cosinor (harmonic) regression. The null hypothesis that there is no circadian rhythmicity (H_0_ = 0) is tested against the alternative hypothesis that a circadian rhythmicity pattern exists (H_A_≠0). The study is considered successful if rhythmicity is detected in some of the measured genes.

#### Analysis for secondary endpoints

Differences in CR and mean gene expression level between subgroups, with respect to sex, age, seasons and physical performance, will be evaluated by CircaCompare [57] to estimate and observe differences between CR, specific to the characteristic desired (mesor, amplitude and phase). The methodology proposed by Parsons *et al*. [57] will be used for exploratory purposes, and circadian curves will be estimated per individual and sampling round. Additionally, correlations between molecular data and physical exercise measurements, as well as for hormonal data (melatonin and cortisol) and activity data (smart-tracker) will be calculated.

#### Missing values and outliers

Missing values will not be imputed. Outliers will be identified by the data management team. According to the decision of the data management team and principal investigator, outliers will be kept in the database or set to “missing”. If a participant did not have an acute (oral or any other) infection at the time of recruitment, but developed an infection during study, the data originated from that round of saliva sampling will not be considered for the data analysis.

#### Development of machine learning analysis pipeline

We will correlate alterations in the gene expression profiles to other measurements collected. The generated data will be used to develop and optimize computational pipelines to accurately assess a parameter for CR-dependent variation in different subgroups of the study participants with respect to sex, age or season. Albeit the results should be further validated in future and larger cohort of studies, the output data can then be further processed to apply a feature selection criterion from the smart tracker data (e.g., heart rate or activity data), which in turn can be correlated with the participants’ other measurements (saliva gene expression data, hormonal data) for assessing their correlation, and used to optimize the machine learning pipelines to train computational models, and predict optimal timings for daily activities such as physical activity. Depending on the data distribution, supervised machine learning algorithms based on linear (e.g., linear discriminant analysis), nonlinear (e.g., classification and regression trees), or complex nonlinear (e.g., Support Vector Machine, SVM) methods will be used.

### Ethics statement

All procedures involving human subjects were approved by Charité—Universitätsmedizin Berlin Ethics Committee (EA2/242/20). The participants were enrolled on a voluntary basis and signed the informed consent approving the analysis of their data in the scope of the study.

### Status of the study and timeline of the study

The study started to recruit upon ethics approval, in 2022. The recruitment and data collection are planned to be carried out until the December 2023.

## Discussion

Circadian medicine has gained significant interest in recent years as the relevance of the circadian system in enhancing overall health and well-being has been recognized by researchers and healthcare professionals. However, there are several challenges hindering the application of circadian medicine into clinical practice. These obstacles include a lack of comprehensive understanding of the intricate mechanisms underlying CR, the need for substantial changes in clinical practices and infrastructure to accommodate circadian-based diagnostics and treatment schedules, and the absence of standardized, easy, and non-invasive approaches to characterize and CR.

Circadian regulation poses optimal time frames for daily activities, examples include meal times [58], hormone secretion [59], and cognitive performance [60]. Physical performance is also among circadian-influenced activities in which previous studies by our group and others pointed to a higher athletic performance reported in the afternoon hours compared to morning hours [34, 61, 62]. The core clock gene, *PER2* impacts the peak timing of exercise whereas the average expression of *BMAL1*, correlates with the muscle tone among healthy, physically active individuals [34].

To further investigate the above-described findings, this study aims to analyze circadian gene expression profiles from saliva samples to characterize CR in different individuals and identify variations based on factors like sex, age, and seasons. The data collected will contribute to a comprehensive understanding of CR profiles and can be correlated with hormonal assessment, questionnaires, and activity tracking to improve sleep, overall health, and optimize daily activities. As a prospective, the study intends to correlate molecular CR profiles with findings related to physical performance and develop computational models to predict optimal exercise timing for individuals with similar circadian rhythm profiles, which can be further validated in future studies. Such findings are not only useful for athletes, but also for patients undergoing physical rehabilitation, as well as for Parkinson’s Disease patients, where physical activity is used to slow down disease progression. The CR profiles collected can also be used to establish a control group of individuals with healthy CR, which may be used for future comparison between CR of individuals with different pathological conditions.

### Limitations of the study

Associated limitations to this study include limited generalizability to broader population groups due to specific inclusion criteria, and potential errors in saliva sample collection by participants despite efforts to minimize them. Nevertheless, this study’s sampling method can assess CR in healthy individuals, making it useful also in clinical and non-clinical settings for future monitoring of disease related to CR disruption, and for adapting daily activities to enhance the CR.

### Dissemination plans

Findings will be disseminated through peer-reviewed publication and via oral and poster presentations at national and international conferences and symposia.
